# Internationally recruited nurses in London: a survey of career paths and plans

**DOI:** 10.1186/1478-4491-4-14

**Published:** 2006-06-26

**Authors:** James Buchan, Renu Jobanputra, Pippa Gough, Ruth Hutt

**Affiliations:** 1 Queen Margaret University College, Edinburgh, UK; 2 The King's Fund, 11 – 13 Cavendish Square, London, W1G 0AN, England, UK

## Abstract

**Background:**

The paper reports on a survey of recently arrived international nurses working in London, to assess their demographic profile, motivations, experiences and career plans.

**Methods:**

A postal survey was conducted in October-December 2004 on a sample of 1000 nurses who were London-based international members of the Royal College of Nursing (RCN). The usable response rate was 40%. Registration data from the Nurses and Midwives Council (NMC) were also analysed.

**Results:**

The Philippines, Nigeria and South Africa were the three most commonly reported countries of training (in total, more than 30 countries of training were reported). Sixty per cent of the nurses from sub-Saharan Africa and more than 40% from South Africa and India/Pakistan/Mauritius were aged 40 or older; the youngest age profile was reported by the Australia/New Zealand/USA nurses. Two thirds of all the respondents indicated that a recruitment agency had been involved in their move to the United Kingdom (UK). Three quarters of the respondents (76%) reported that they were required to complete a supervised practice course/period of adaptation in the UK in order to be eligible to practice as a nurse in the UK. Two thirds (69%) of respondents were working in NHS hospitals in London, 13% were working in the private sector hospitals and 10% were working in private sector nursing homes. Most of the nurses reported they were the major or sole wage-earner contributing to household income. More than half of the respondents (57%) reported that they regularly sent remittances to their home country. The majority of respondents (60%) indicated that they planned to stay for at least five years, but just under half (43%) also reported that they were considering a move to another country.

**Conclusion:**

One critical issue for UK policy-makers is to determine if internationally recruited nurses will stay on in the UK, move back to their home country, or move on to another. That these nurses have made at least one international move means they are likely to have the propensity to move again. As such, retention efforts in the UK will have to take account of their career aspirations.

## Background

This article reports on the results of a survey of international nurses working in London [1]. The United Kingdom (UK) has been active in the international recruitment of health professionals in recent years. There is devolved government in the UK. England, the largest country, has been the most active recruiter. As a result of planned and funded expansion of the National Health Service (NHS) in the UK, there has been a need to rapidly "scale up" the numbers of nurses and doctors working in the NHS. The UK governments have been successful in increasing the numbers being trained, and in attracting back "returners" who were not practising, but there has also been an explicit policy emphasis on international recruitment as a method of increasing the NHS workforce [2].

The issue of international recruitment and migration of health workers has generated significant media attention in the UK and elsewhere, primarily because of concern about the impact on source countries in the developing world, but much of the reportage has been anecdotal. There has been relatively little primary research on this issue, and most that has been published either focuses on assessing the impact of international recruitment in terms of trends in cross-border flows of health workers, or reports on small-scale focus-group interviews [3,4]. There is much written on the "push" and "pull" factors stimulating health professionals to move and migrate, but little is evidence-based, and not much is known about the profile, motivations and plans of the health professionals who have actually made an international move.

The main objectives of the paper are to report a survey of "recently arrived" international nurses working in London, to assess their demographic profile, motivations, experiences and career plans, to give an insight into why they have come to the UK and their future intentions. In order to contextualize these findings, the paper also outlines the overall trends in numbers of nurses coming to the UK and the policy context in which international recruitment activity has been conducted.

Previous analyses of unpublished post code (zip code) data suggest that a much higher proportion of international nurses work in London than elsewhere in the UK [5]. This is likely to be partially a reflection of the general trend for migrants to gravitate towards London as the major port of entry to the UK, and also that NHS employers in London report much higher levels of job vacancies than elsewhere in the country. Nurse vacancy rates in London are twice the national average: 3.8% of registered nursing posts in London are vacant for three months or more, compared to an average in England of 1.9% [6], and London-based employers have tended to be particularly active in using international recruitment to fill vacancies.

## Methods

To provide background data on trends in inflow of nurses to the UK, annual registration data from the Nurses and Midwives Council (NMC) were analysed. All nurses working in the UK must be registered with the NMC; new "home" educated and international registrations are identified separately on the register, so it is possible to assess the relative size of each source.

The main study of international nurses in London was based on a postal survey. A postal questionnaire was sent to the home address of a random sample of 1000 nurses who were international members of the Royal College of Nursing (RCN) and reported a London address. The RCN is the largest professional association of registered nurses in the UK, representing more than half of all nurses working in the UK. These nurses had become members of the RCN no more than two years before the survey was conducted.

The questionnaire was first pre-piloted to check on its cultural and language relevance. A full postal pilot of 100 nurses was conducted in August-September 2004, after which minor modifications were made to the questionnaire. The full postal survey with the final version of the questionnaire was conducted in October-December 2004.

Questionnaires were mailed to the home address of a random sample of RCN international nurses in London in October 2004 with one reminder being sent in November. There were 60 undelivered or postal returns indicating addressees had moved, and 380 usable returns, giving a response rate of 40% (380/940).

## Results

### Trends in inflow of nurses to the UK

**Figure 1:**
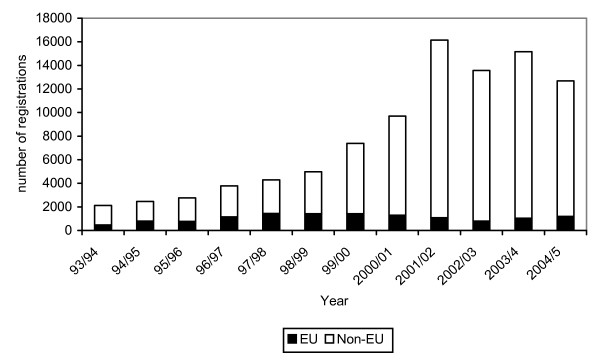
Admissions to the UK nurse register from EU countries and other (non-EU) countries, 1993/94 – 2004/5.

In the period between 1999 and 2003 there was rapid growth in the numbers of nurses from other countries registering to practise in the UK. While the annual number of international nurse registrants entrants has now declined, it remains at historically high levels. In the year up to March 2005, more than 12 000 nurses were admitted to the UK from all overseas countries (Figure 1).

Most of the growth in inflow of nurses to the UK has been from countries outside the European Union (EU). The four most important source countries in 2004/5 were India, the Philippines, South Africa and Australia. The vast majority of nurses coming to the UK are from English-speaking countries of the Commonwealth, or from the Philippines.

In total, between April 1997 and March 2005 there was an aggregate total of more than 80 000 overseas nurses admitted to the UK register. The relative contribution of international nurses to staffing growth in the UK has risen significantly. In the early 1990s: overseas countries were the source of about one in 10 nurses entering the UK register. In recent years, overseas countries have, on average, contributed about four out of 10 of the annual number of new nurse entrants to the UK register [2].

While NMC data can assist in tracking overall trends in the numbers of international nurses becoming eligible to practice in the UK, there are no complete and accurate published data available on where these nurses are located within the UK, if they are actually practising, or what type of work they are undertaking. Overall, about three quarters of all working nurses in the UK are employed in the NHS, the remainder working in the independent (i.e. private) sector, in nursing homes and in the relatively small independent acute hospital sector [2]. Both the NHS and the independent sector have been active in recruiting internationally, but it is not known in any detail where the level of use of international nurses is most prominent. The NHS in England does not record how many international nurses it employs, despite a recent recommendation by the House of Commons Select Committee on Migration [7].

### Findings of the survey of international nurses

The decision to focus the survey of international nurses on those working in London was based on an understanding that there was a high concentration in the capital. The use of the RCN membership data base also enabled the questionnaire to be targeted both at nurses working in the NHS and in the independent sector, as RCN membership extends to both sectors.

### Demographics of survey respondents

The objective of the survey had been to target nurses who had arrived in the UK within the last few years. While it was not possible to determine this from the available RCN membership information, the date of first joining the RCN was used as a proxy when creating the sample. Most respondents (77%) reported that they had first arrived in the UK since 2001, and nearly all the nurse respondents (96%) reported that they had first arrived in the UK since 2000. All but one reported that they had first worked as a nurse in the UK since 2000. The survey respondents therefore represent a population that had spent four years or less in the UK. Given the significant increase in international nurse arrivals in the UK since 2000, shown in Figure 1, this is not surprising.

**Figure 2:**
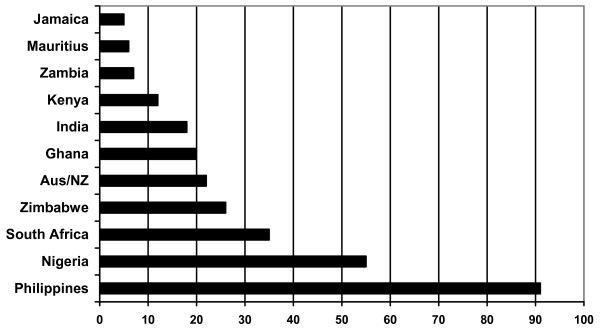
Main reported source countries.

The 380 respondents comprised a population with more than 30 different countries of training. The Philippines, Nigeria and South Africa were the three most commonly reported countries of training (Figure 2). A range of other countries in Africa, Asia, North America and Europe were reported, but in all cases the numbers were relatively small. In recent years, the NMC has registered nurses from 70 or more countries.

Most respondents reported that their country of training was the same as their previous country of location, with the exception of some Filipino and Indian nurses who reported that they and previously been working in the Middle East.

For the purposes of country and regional comparison, some of the data analysed in this paper are presented in regional aggregate form, in five regional categories, by country of training: the Philippines, India/Pakistan/Mauritius; South Africa; other sub-Saharan African countries; Australia/New Zealand/United States. These five regional categories account for 349 of the total of 380 respondents.

**Figure 3:**
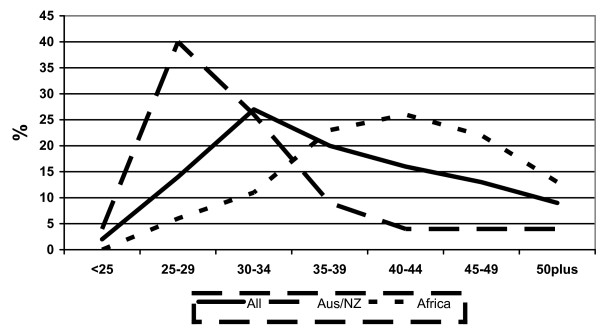
Age profiles: all international nurses, and those from Australia/New Zealand and sub-Saharan Africa.

While there is often an assumption that younger nurses are more likely to be internationally mobile, the age profile of respondents varied markedly by regional grouping. Sixty per cent of the nurses from sub-Saharan Africa and more than 40% from South Africa and India/Pakistan/Mauritius were aged 40 or older; the youngest age profile was reported by the Australia/New Zealand/USA nurses, with more than 60% being aged 34 or younger. Figure 3 highlights the significant variation in age profile between the relatively "younger" Australia/New Zealand/USA group and the older profile of nurses from sub-Saharan Africa.

Nursing is mainly a primarily a female occupation in most countries. Over 90% of UK-trained nurses are female [8]. There was a higher proportion of male nurses in the international nurse respondents, with 84% being female. Two thirds (66%) of respondents reported they were married. Three quarters of respondents (76%) who reported that they were married or had a partner also reported that they were currently living with their spouse/partner in the UK; one quarter (24%) reported that their spouse/partner was living in their home country.

**Figure 4:**
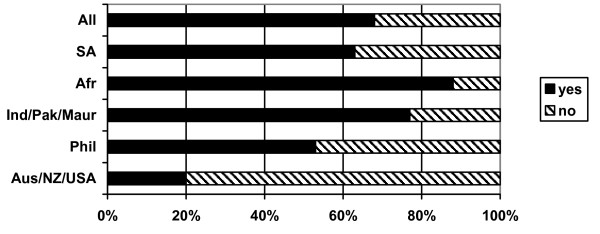
Do you have children? (response by main regional groupings).

Two thirds of respondents (66%) reported having children (Figure 4). Most respondents from sub-Saharan Africa (88%), India/Pakistan/Mauritius (77%), South Africa (63%) and the Philippines (53%) reported having children. Only 22% of Australia/New Zealand/USA respondents had children. Of these respondents, 61% had children living with them in the UK and 39% reported children living in their home country. Some respondents reported having children both in the UK and in their home country.

Nearly all the respondents (92%) are qualified and registered to practise in general adult nursing: 10% are registered to practise in mental health nursing, small numbers reported registration as learning disabilities nursing, children's nursing or midwifery. Some respondents are registered to practise in more than one field.

### Coming to the UK

**Figure 5:**
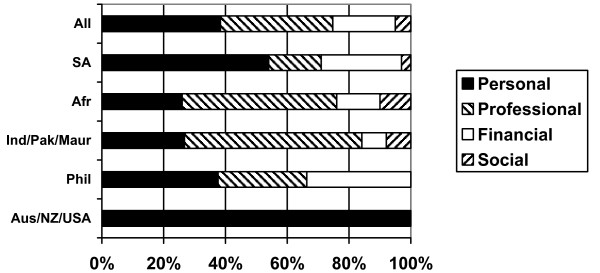
Type of factor most influencing decision to come to the UK.

Respondents were asked to report the reason that had most influenced them to decide to come to the UK. The key results are shown in Figure 5. The responses highlight some variation by region of origin. All the Australia/New Zealand/USA nurses indicated that the main reason that they were in the UK was personal, linked to travel and experiencing a different way of life. The results from the other regional groups question the assumption that nurses are moving only for financial reasons: many report that the factor that most influenced them to move was professional development. Some nurses from Africa and India/Pakistan/Mauritius reported social reasons as being the main driver – linked primarily to joining family already in the UK. No nurses from the Philippines reported this reason for coming to the UK. This is not surprising, as there is no history of migration from the Philippines to the UK and the post-colonial ties that exist between the UK and anglophone Africa and Asia are absent.

Two thirds of all the respondents indicated that a recruitment agency had been involved in their move to the UK. Relatively fewer nurses who had previously been located in sub-Saharan Africa had made use of an agency, but nearly all Philippines-based nurses (96%), South African nurses (83%) and most nurses who had been based in the Middle East and in India/Pakistan/Mauritius reported that a recruitment agency had been involved in their move. Filipino nurses were most likely to report that the agency was based in their home country (i.e. Philippines); for nurses from the other regional groups, the agency was more likely to have been international or based primarily in the UK.

Nearly three out of every four nurses (72%) who reported using an agency had to pay for at least part of the services provided by the agency (i.e. the recruiting employer was not covering all the recruitment/registration/travel costs). Filipino (74%) and India/Pakistan/Mauritius nurses were most likely to report that they had paid. Most Australia/New Zealand/USA nurses (78%) reported they did not have to pay for any services provided by agencies. The most commonly reported payments were direct fees to the agency; adaptation fees to the Nurses and Midwives Council in the UK, and transport fees to travel to the UK to take up their job.

### Supervised practice/adaptation

**Figure 6:**
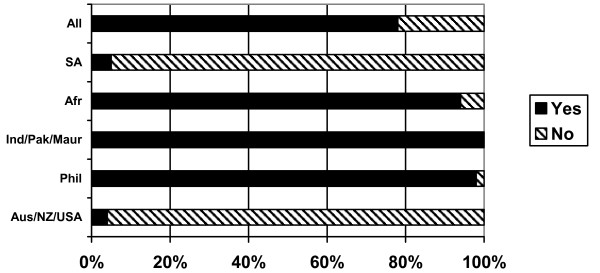
Did you have to complete supervised practice/adaptation to practice in UK? (response by main regional groupings).

Three quarters of the respondents (76%) reported that they were required to complete a supervised practice course/period of adaptation in the UK in order to be eligible to practise as a nurse in the UK. The requirement to undertake supervised practice/adaptation varied significantly depending on country of training (Figure 6). Nearly all Australian/New Zealand/USA and South African nurses reported that they were not required to undertake a course prior to registration to practise in the UK, but all nurses from India/Pakistan/Mauritius and nearly all from the Philippines and sub-Saharan Africa reported that they had to take a course/period of adaptation.

In the majority of cases, this course was reported to have been taken while the nurses were working for private sector nursing homes (nurses from India/Pakistan/Mauritius and sub-Saharan Africa) or in NHS hospitals (nurses from the Philippines).

### Current employment

**Figure 7:**
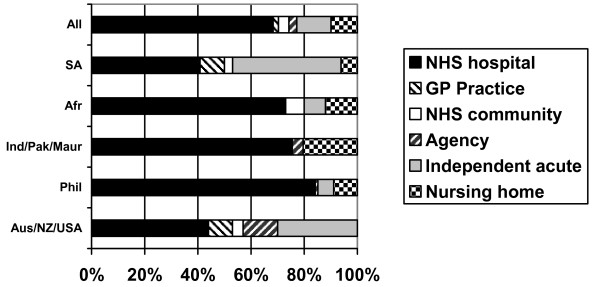
Current employer, main job (response by main regional groupings).

Two thirds (69%) of respondents were working in NHS hospitals in London, 13% were working in the private sector hospitals and 10% were working in private sector nursing homes (Figure 7). Very few respondents were working either for general practices or in NHS community nursing. In part this may be explained by the fact that some NHS community nursing posts require post-basic professional qualifications that are not available in other countries. Filipino nurses were most likely to be working in NHS hospitals; as were the majority of nurses from other regions apart from sub-Saharan Africa (where many were working in the private sector), South Africa (where 40% reported they were working in the private hospital sector) and Australia/New Zealand/USA (where some reported they were working directly for nursing agencies).

**Figure 8:**
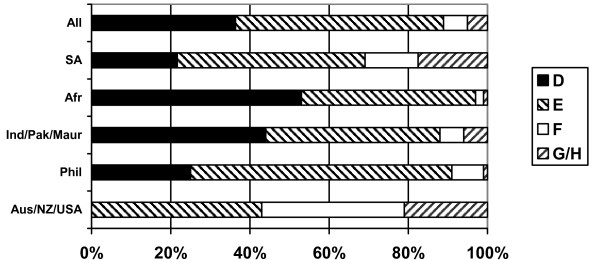
Reported NHS clinical grade, by main regional groupings.

More than half of the respondents (57%) had already made one change of employer since beginning work as a nurse in the UK. The main direction of employment mobility had been from the private sector and nursing home sector to the NHS. Of those who have made a move, three quarters (75%) report that their first employment in the UK was as a nurse in the private/independent sectors.

### Pay and grading

At the time of the survey, all NHS nurses working in clinical practice were paid according to a single national pay/grading system ("clinical grading"). This system is based on grading structure from grade "A" (lowest) to grade "I" (highest). Three quarters of respondents reported that they were paid on the NHS clinical grading system. Some private sector employers also use the clinical grading system. Data on reported clinical grade enable an assessment of variation in pay rates by different regional grouping.

Nearly all the respondents who were paid according to clinical grading reported that they were paid on either clinical grade D (36%) or grade E (51%). These are the two main grades for staff nurses (the primary job category for registered nurses). There was evidence of variation by region of training: more than half of the nurses from sub-Saharan Africa (53%) were graded at the lower level of D, as were nearly half of the nurses from India/Pakistan and Mauritius. Two thirds (65%) of Filipinos reported that they were graded at the higher level of grade E. None of the nurses from Australia and New Zealand reported that they were paid at grade D: more than half of this group were paid at grade F or above. Similar variation in grading outcome has been reported in other, more recent, surveys of UK nurses [9].

**Figure 9:**
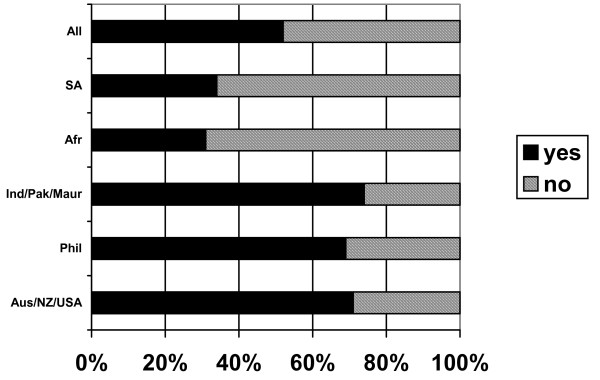
Do you consider your clinical grade to be appropriate, given your role and responsibilities? (response by main regional groupings).

Respondents were asked to indicate if their current clinical grade was appropriate, given their role and responsibilities (Figure 9). Just over half (53%) of those who were graded indicated that they believed their grade was appropriate, but this dropped to only 31% of nurses from sub-Saharan Africa and 34% of nurses from South Africa.

**Figure 10:**
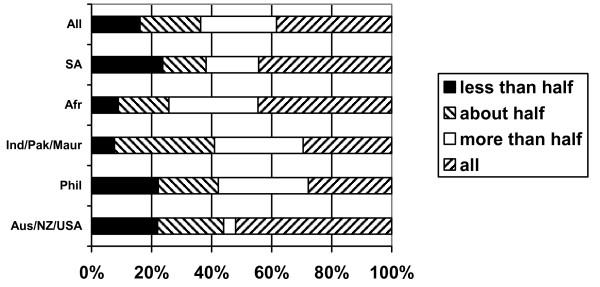
What % of total household income do your earnings as a nurse represent? (response by main regional groupings).

Most of the nurses were the major or sole "breadwinner" contributing to household income. One third (37%) were contributing all of the household income, a further quarter (25%) contributed more than half, and a further one in five (20%) contributed about half (Figure 10).

**Figure 11:**
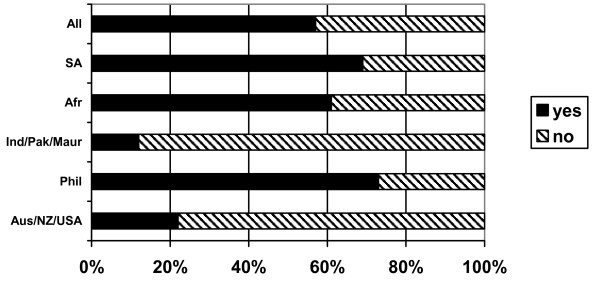
Do you regularly send home money (remittances) to your home country? (response by main regional groupings).

More than half of the respondents (57%) reported that they regularly sent remittances to their home country, but the pattern of remitting varied significantly by regional grouping (Figure 11). Three quarters of Filipino nurses (73%) regularly remit money home, as do more than half of nurses from sub-Saharan Africa and from South Africa. Nurses from Australia/New Zealand/USA and India/Pakistan/Mauritius were much less likely to report that they were remitting money. In the former case this may be linked to the fact that they are more likely to be single, and more likely to be planning only a short stay in the UK (see below). In the latter, it may be linked to the fact that these nurses have their families with them in the UK. Nurses from South Africa and the Philippines were most likely to report that they remitted a high proportion of their income – in both cases, about half of respondents were remitting either between 26% and 50% or more than 50% of their income.

**Figure 12:**
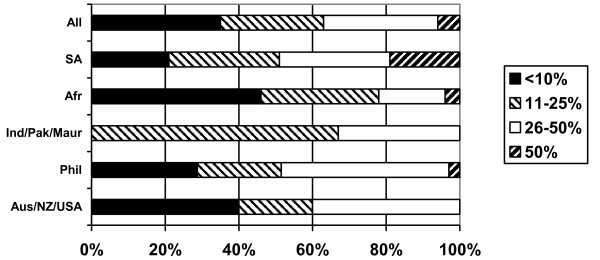
Proportion of earnings remitted home (respondents who reported remitting).

The average full-time pay for a nurse in the UK in 2004 was approximately GBP 24 500 [10] (nurses in London earn more because of a regional supplement).

### Career plans

**Figure 13:**
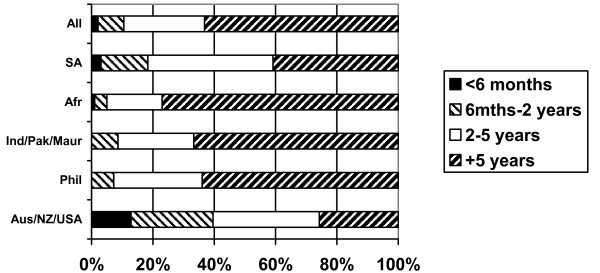
How long do you plan to stay in the UK as a nurse? (response by main regional groupings).

Respondents were asked to indicate how long they planned to remain in the UK as a nurse (Figure 13). The majority (60%) indicated that they planned to stay for at least five years, with a further quarter (25%) indicating that they planned to stay between two and five years. Australia/New Zealand/USA nurses were least likely to be planning to stay long-term and proportionally more South African nurses reported planning to stay for two to five years than for longer periods.

Most respondents (83%) require a work permit to work in the UK and nearly all (91%) indicated that if their permit was extended they would wish to stay longer in the UK.

**Figure 14:**
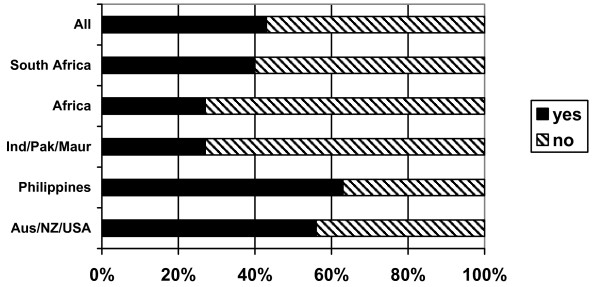
Are you considering a move to another country? (response by main regional groupings).

Respondents were also asked if they were considering a move to another country. Just under half (43%) reported that they were considering a move (Figure 14). Nearly two thirds of Filipinos (63%), more than half of Australia/New Zealand/USA nurses and 40% of South African nurses were considering a move. Nearly all the Filipino nurses (83%) who were thinking of moving reported that they were considering moving to the USA, while Australia/New Zealand/USA nurses and nurses from South Africa were most likely to be considering moving "back home". Overall the USA was the most often reported potential destination, cited by more than half of the potential movers; Australia was the next most commonly reported possible destination.

One third of the respondents (32%) had been contacted by a recruitment agency within the last six months and offered work outside the UK, including half of all the Filipino nurses (who were mainly being offered work in the USA).

## Discussion of survey results

The survey of several hundred international nurses working in London has provided a detailed picture of their demographic profile, their motivations for working in the UK, their career plans and also some information on their pattern of remittances. The survey provides more insight into these issues than has been available before, and highlights a range of key issues which have implications both for broader-based UK national reliance on international recruitment of nurses and local practice in retaining and motivating these nurses and treating them fairly.

The first point to note is that the sheer diversity of the range of countries from which nurses are being recruited has implications for policy and practice. The broad range of source countries for UK-based international nurses has been obvious in the Nurses and Midwives Council registration data from recent years, but this current survey highlights the extent to which different countries of training can be related to different demographic profiles and reported career intentions. While it can be misleading to generalize based on source country or grouping of source countries, there are marked variations in terms of respondent demographic profile and in terms of responses to some questions from some of the regional subgroupings. To focus policy attention or practice on all internationally recruited nurses as being "the same", but somehow "different" from all UK-educated nurses, is at best an oversimplification of a complex situation and could be a dangerously misleading approach.

While it can be misleading to focus on generalities, it is clear that different types of internationally mobile nurses can be delineated within the survey: the young "backpacker" nurse from Australia or New Zealand who is planning a relatively short stay in the UK has a different range of priorities and objectives than a Filipino nurses remitting money back to her extended family (and perhaps considering a move to the USA); both are different from an older South African nurse taking the opportunity of a few years in the UK for professional development before planning to return home.

Several key themes do emerge, which have implications for policy and practice in the UK. The broad age profiles of nurses, particularly the "older" profile from Africa, counters the assumption often being made that it is only young nurses who are internationally mobile. Some of the mobile nurses are aged in their 40s or 50s and have many years' clinical experience. This reinforces the point that the impact of emigration on sub-Saharan countries is not just about numbers, it is about a loss of experienced staff.

The demographic data also revealed that many nurses have their partner and/or children with them in the UK, which highlights that not all have travelled leaving their spouse and other close relatives "at home": for some, in a sense, home has travelled with them. However, one in three nurses with children reports that they have left children in their home country.

It was also evident from the responses to the survey that financial reasons are not always the reported primary driver for international nurses to be in the UK; many have been attracted to the UK for a variety of other reasons, primarily for professional development reasons or to take the opportunity to travel. These are self reported reasons so must be treated with some caution, but they do highlight a more complex reality than that based on the assumption that money is always the only, or main, driver to migrate.

The central role played by recruitment agencies in both stimulating and facilitating international recruitment was highlighted in the survey. Two thirds of the international nurses working in London reported that a recruitment agency had been involved in their move to the UK – and most had to pay for some of the services provided by the agency. Some of the nurses reported that they had been provided with misleading information by agencies about their pay and working conditions in the UK. Recruitment agencies providing staff to the NHS have recently been brought within the remit of the Department of Health Code practice (discussed below).

The regulatory requirements for nurses entering the UK are stringent and based on an assessment of each applicant. Most international nurses from sub-Saharan Africa, the Philippines and India/Pakistan/Mauritius were required to complete a supervised practice course/or period of adaptation in order to practise in the UK; most had done so in private sector nursing homes, and some nurses from sub-Saharan Africa reported that they had to pay for their adaptation, or received no pay during that period. While these regulatory requirements are in place to maintain standards and for public protection, the response from some of the nurses revealed that they believed they had been exploited during their application and entry process.

The survey evidence on the levels of remittances being sent, although limited, does add new information on this important but under-explored issue. It is important to note that most of the nurses reported that they were the sole or main contributor to family income. More than half of the nurses reported that they regularly remitted money to their home country; nurses from the Philippines and South Africa were more likely to remit a higher proportion of their income – half of each group regularly remitted a quarter or more of their income. This represents a significant flow of money.

The UK policy context in which the survey evidence must be examined is codified within a so-called "ethical" approach. Recruitment of nurses from the developing world has been controversial, and the Department of Health in England has attempted to limit the potential negative impact. It first established guidelines in 1999 [11], which required NHS employers not to target South Africa and the West Indies, and then introduced a Code of practice of international recruitment for NHS employers (in 2001) [12], which was strengthened (in 2004) [13]. This Code requires NHS employers not to actively recruit from developing countries unless there is a government-to-government agreement that active recruitment is acceptable. At the time of writing, such agreements exist only with China, India, and the Philippines – all other developing countries are effectively designated as "no go" areas for NHS recruiters. So far, there has been little active recruitment of nurses from China.

The Code applies to NHS employers, to "preferred provider" recruitment agencies and to private sector employers if they are providing NHS-funded care. The overall impact of the Code is difficult to monitor and assess because of because of an absence of NHS-specific data on numbers of nurses recruited and employed. However, this survey of London-based international nurses clearly demonstrated that many nurses were recruited initially by private sector nursing homes in the UK but moved quickly to the NHS on completion of their adaptation period in the UK. NHS employers in London were the end beneficiaries of private sector "back door" recruitment from countries that were on the NHS "banned " list of developing countries. This does not contravene the NHS Code on international recruitment, but helps explain why there continues to be an annual inflow of several thousand nurses to the UK from developing countries on the list.

The issue of the efficiency and effectiveness of international recruitment rests partially on how long international recruits are retained within the NHS. The survey highlighted that many of the international nurses were thinking about a long-term commitment to the UK (especially those from sub-Saharan Africa and the Indian subcontinent), others were planning to go home (especially nurses from Australia, New Zealand and South Africa); but many were also considering moving on (primary destination the USA), stimulated by recruitment agency contact (this especially the case for nurses from the Philippines).

### Limitations of the study

The survey was based only on RCN members, who represent the majority, but not all, working nurses in the UK. Nurses who were in the UK for only a short period may be less likely to join the professional association, so may be underrepresented in the study. Nurses from countries without a culture or tradition of joining a professional association may be underrepresented in the study. The survey had an acceptable response rate, but it was not feasible to identify reasons for non-response. Fewer respondents provided information on remittances than on the other topics covered by the questionnaire; this may reflect a greater reluctance on the part of respondents to provide information on financial details than on other subjects.

NMC data provide information on all nurses accepted onto the UK register, it does not necessarily mean that all these nurses are actually in the UK.

The focus on London provides a detailed insight into the profile and motivations of nurses in the capital; given the relatively higher proportion of international nurses working in London; the results should not be taken to be representative of all international nurses working in other parts of the UK. The sample sizes for some source countries are too small to be taken as representative of all nurses recruited from these countries.

## Conclusion

One critical issue for UK policy-makers is to determine whether internationally recruited nurses will stay on in the UK, move back to their home country, or move on to another: Is London a gateway or a revolving door? The survey provides a mixed picture. The majority of the nurses were considering a long-term stay (five years or more) in the UK. In part this depended on the provision of an extension to their work permit. Many nurses were also considering the possibility of moving on to another country; in particular, many Filipino nurses were thinking of a move to the United States. That these nurses have made at least one international move means they are likely to have the propensity to move again. As such, retention efforts in the UK will have to take account of their career aspirations.

## Competing interests

The author(s) declare that they have no competing interests.

## Authors' contributions

JB conceived of and coordinated the study, participated in its design and analysis and drafted the manuscript; RJ participated in design, managed the survey, conducted the analysis of data and contributed to drafting; PG contributed to drafting; RH participated in analysis and drafting. All authors read and approved the final manuscript.
